# Chicago Dental Society Organization

**Published:** 1864-04

**Authors:** 


### CHICAGO DENTAL SOCIETY ORGANIZATION.

At a meeting of the Chicago Dental Society on Monday Evening, March 14th, some of the members desiring to have the proceedings of the previous meeting published, Dr. Cushing offered the following, which was adopted:

*Resolved,*—That the Secretary be requested to furnish the daily papers of this city, the *Dental Cosmos and the Dental Register of the West*, a condensed report of the proceedings of the society at its last meeting, so far as relates to its organization and the election of officers.

In accordance with the resolution, the following report is presented:

On Monday Evening, February 8th, the following named Gentlemen of this city, met at S. S. White's Dental Depot, for the purpose of forming a Dental Society: Dr. E. W. Hadley, James C. Dean, John C. Fuller, Wm. Albaugh, W. W. Allport, J. H. Young, G. H. Cushing, L. R. Haskell, S. B. Noble, E. W. Sawyer, L. Bush and J. Ward Ellis. Dr. E. W. Hadley, the oldest resident Dentist of the city, was elected temporary Chairman, and E W. Sawyer, temporary Secretary; and the Chicago Dental Society was organized, by adopting a constitution and by-laws, and electing the following officers:

*President*—Dr. E. W. Hadley,

*Vice Presidents*—Dr. J. H. Young, Dr. L. Bush.

*Recd. and Cor. Secy.*—Dr. E. W. Sawyer.

*Treasurer*—Dr. J. C. Dean.

*Executive Committee*—Dr. L. P. Haskell, Dr. S. B. Noble, Dr. Wm. Albaugh.

*Librarian*—Dr. W. W. Allport.

Dr. S. S. White, of Philadelphia, and S. R. Bingham of this city, were elected honorary members. At a meeting of the Chicago Dental Society, Monday Evening, March 14th, the following Gentlemen of this city were elected members of the Society: Dr. A. W. Freeman, B. M. Baker, A. J.

Harris, E. A. Bogue, T. P. Abell, E. R. E. Carpenter, J. A. Kennicott, H. Hall, T. Fay, J. Deschaur, J. W. Smith, and W. A. Stevens. And the following honorary members, proposed by Dr. Allport, were elected: Dr. J. H. McQuillen, of Philadelphia, Pa.; Wm. Pierce, of Philadelphia, Pa.; J. Taft, of Cincinnati, O.; J. Taylor, of Cincinnati, O.; J. Richardson, Terre Haute, Ind.; A. Hill, Norwalk, Conn.; Geo. Watt, Xenia, O.; W. H. Allen, New York City.; Dr. Nones, Del.; N. B. Kingsley, of New York City.; H. B. McKillops, St. Louis, Mo.; E. W. Shaulding, St. Louis, Mo ; H. E. Peebles, St. Louis, Mo.; D. W. Perkins, of Milwaukee, Wis.; and E. F. Willson, of Chicago.

Dr. Hadley delivered an excellent address, showing the benefit and power of combined effort, and the exercise of Christian principles, "and the greatest of these is charity." He spoke of the difficulties that were encountered by the profession twenty years ago, and his own efforts to overcome them; and closed with the wish and the hope that this society, so happily begun, may continue "*Not for a day, but for all time.*" After Dr. Hadley's address, Dr. Allport read a paper before the Society, which ought to be read by every one in the community, showing the absolute necessity of keeping the teeth clean, in order to prevent their becoming disceased, and gave it as his opinion that three-fourths of the teeth now injured by disease could have been kept healthy by proper care in this respect on the part of the patient. He endeavored to impress upon our minds that it is the duty of the true Dentist not only to perform all operations faithfully, but to endeavor to instruct the people how to avoid the cause. The Society voted that a copy of Dr. Hadley's address, and the paper read by Dr. Allport be furnished the *People's Dental Journal*, the *Dental Cosmos* and the *Dental Register of the West.* Voted to adjourn to the second Monday in April.

